# Validation of the Turkish version of the Obstetric Quality-of-Recovery score 11 (ObsQoR-11T) after cesarean delivery

**DOI:** 10.1186/s12955-022-02073-y

**Published:** 2022-11-28

**Authors:** Gökhan Ozkan, Umut Kara, Mehmet Emin Ince, Ozhan Ozdemir, Mustafa Ulubay, Serkan Senkal

**Affiliations:** 1 Department of Anaesthesiology and Reanimation, Gülhane Training and Research Hospital, University of Health Sciences, 06010 Ankara, Turkey; 2 Department of Gynecology and Obstetrics, Gülhane Training and Research Hospital, University of Health Sciences, Ankara, Turkey

**Keywords:** Cesarean delivery, Cross-cultural comparison, Patient-reported measures, Quality of recovery, Recovery, Postoperative

## Abstract

**Background:**

To translate and validate the psychometric characteristics of a Turkish version of the Obstetric Quality-of-Recovery score 11 tool used to measure post-cesarean delivery recovery in Turkish-speaking patients.

**Methods:**

After the original English version of the Obstetric Quality-of-Recovery score 11 tool was translated into Turkish; it was psychometrically validated to assess the post-cesarean delivery quality of recovery. Validity, reliability, and feasibility were investigated. The Obstetric Quality-of-Recovery score 11 tool was administered to Turkish-speaking patients on postoperative day 1. On postoperative day 1, a global health visual analog scale was used to assess the patient's perceived global recovery.

**Results:**

One hundred and eighty-six patients completed their questionnaires, providing a completion rate of 97.38%. The Spearman rho (ρ) correlation coefficient between the Obstetric Quality-of-Recovery score and global health visual analog scale (0–100 points) was 0.850 at postoperative day 1 following surgery (*P* < 0.001). Internal consistency, measured using Cronbach’s alpha, was 0.822. The split-half coefficient was 0.708. The Obstetric Quality-of-Recovery score differed significantly between the emergency and elective cesarean delivery groups (80 (41–104) vs. 83.3 (51–102); *P* < 0.05). The test–retest reliability of the Obstetric Quality-of-Recovery score items was more than 0.6 in 82% of cases, indicating good repeatability and reliability.

**Conclusion:**

The Obstetric Quality-of-Recovery score 11 is a valid and reliable tool to measure the post-cesarean quality of recovery in Turkish-speaking patients. The psychometric properties of the Turkish version of the scale to measure the post-cesarean quality of recovery were similar to those of the seminal English version.

## Introduction

Recovery after cesarean delivery (CD) is a multidimensional and complex process influenced by a variety of factors, such as patients, obstetric procedures, and anesthetic characteristics. The majority of research on CD recovery has focused on physiological parameters, including pain, nausea/vomiting, recovery of bowel function, length of hospital stay, recovery timeframes, and the occurrence of adverse events such as poor outcome and mortality [1, 2]. There is an increasing focus on the patient-perceived quality of recovery (QoR). Patient-reported outcome measures (PROMs) can be used to assess the patient's perspective [3].

The Quality of Recovery-40 (QoR-40) score was developed in 2000. It is now widely used. It has also been successfully translated and validated in the Turkish language [4]. The Obstetric Quality-of-Recovery (ObsQoR-11) score, derived from the QoR-40 scale, was developed to assess recovery in the first 24 h after CD [5]. The ObsQoR-11 scale is a composite patient-reported outcome measurement of the quality of recovery that evaluates four underlying factors as follows. Physical comfort and pain are represented by Factor 1. Factor 2 reflects both physical independence and mental well-being. Factor 3 represents physical independence, whereas Factor 4 supplements Factor 1. The ObsQoR-11 scale provides a score ranging from 0 to 110, with a high score indicating a good recovery.

The ObsQoR-11 tool has evolved to the ObsQoR-10 questionnaire in the 2020 [6, 7]. One item was created by combining the items for moderate, and severe pain. The ObsQoR-10 tool hadn't been published when our study was in the design, planning, and protocol approval phases.

This study aimed to develop the Turkish version of the ObsQoR-11 (ObsQoR-11T) through a translation and cultural adaptation process, and to evaluate the validity and reliability of the ObsQoR-11T for Turkish women who had an elective and emergency cesarean delivery. The authors hypothesized that the ObsQoR-11T would have comprehensive validity and reliability, similar to the original English version.

## Methods

### Patient selection

This prospective observational cohort study of term women undergoing CD was approved by the Ethics Committee of Gülhane Education and Research Hospital, Turkey (No. 2021/506), and registered with ClinicalTrials.gov (NCT04744311, February 8, 2021). Written informed consent was obtained from all the participants. The study was conducted in line with the principles of the Declaration of Helsinki [8]. All methods were carried out following the Strengthening the Reporting of Observational Studies in Epidemiology guideline [9]. Patients who underwent surgery at the hospital between January 2021 and August 2021 were enrolled.

Women aged ≥ 18 years who underwent CD at ≥ 37 weeks of pregnancy, and were able to read and speak Turkish, were included in the study Patients who were lack of fluency in Turkish, inability to read or understand written Turkish and inability to obtain written informed consent due to neuropsychiatric disorders such as schizophrenia, mental retardation, seizures with eclampsia and addiction were excluded from the study that may bias the ObsQoR-11T measurements.

### Development of the ObsQoR-11T

**Fig. 1:**
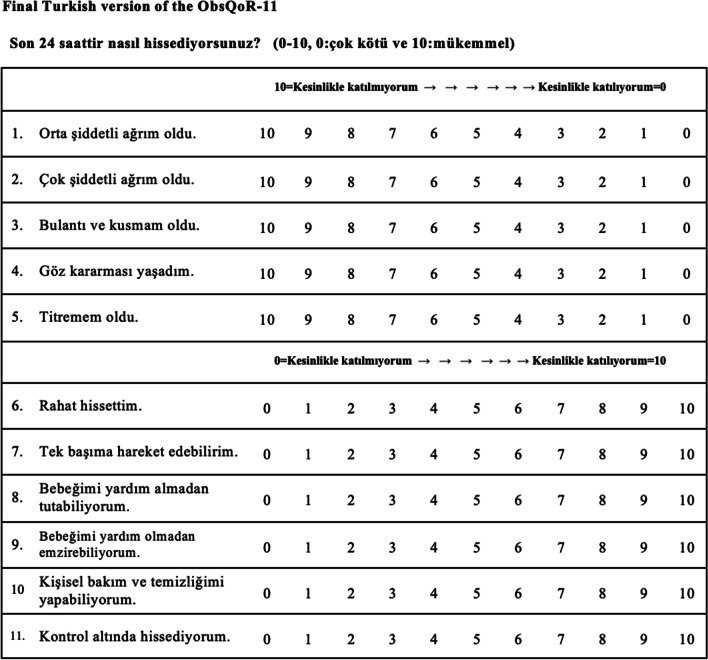
Final Turkish version of the ObsQoR-11 scale

Permission was received from the author of the original English language version of the ObsQoR-11 scale. The translation technique was performed as per the recommendations of Beaton and Bullinger [10]. First, two authors (UK and MEI) translated the ObsQoR-11 into Turkish with reference to the Turkish version of the validated QoR-40 (QoR-40T) [4]. A temporary Turkish version of the ObsQoR-11 was agreed upon, which was then back-translated by a third person (co-author SŞ; healthcare experience in the USA and Turkey). Subsequently, a consensus was made regarding the ObsQoR-11T. The ObsQoR-11T was then tested using a daily working list with a simple randomly selected cohort of ten nurses. All ObsQoR-11T questions were confirmed to be comprehensible. The final ObsQoR-11T is shown in Fig. 1.

### Data collection

Informed written consent was obtained from each patient before surgery. Demographic characteristics were recorded preoperatively. Intraoperative features were obtained from electronic and written patient records. On the morning of the elective scheduled CD, the ObsQoR-11T scale was explained to the patient and consent gained. Before an emergency CD, while the patient was in the preoperative room consent gained. The ObsQoR-11T was administered at 24th hour following CD. At the 25th hour, a computer-assisted randomization program (random.org) was used to determine a random subset of 20 patients prior to complete the ObsQoR-11T scale once again. The researchers, who were members of the perioperative care team, were on hand to assist the patients the ObsQoR-11T. The time required to complete each ObsQoR-11T scale was recorded. The general well-being was measured with a 100-mm global health visual analog scale (VAS) with the ObsQoR-11T questionnaire. The VAS scale ranges from 0 to 100 mm, indicating poor to best possible recovery. The ObsQoR-11T scale and the VAS scale were administered using self-assessment, with assistance as required.

Our institutional neuraxial anesthesia regimen includes intrathecal administration of hyperbaric bupivacaine 12–15 mg, with fentanyl 15 mcg, via a single spinal injection. There is no routine approach for general anesthesia and is dependant on the anesthesiologist, For postoperative analgesia, patients regularly received paracetamol 1 g four times daily unless contraindicated. Patients are also routinely prescribed I.M. diclofenac 75 mg as required after surgery. Intravenous ondansetron 4 mg as required were also prescribed unless contraindicated. Generally, 6 h after the spinal anesthesia, patients are encouraged to mobilize, and 8 h after, a trial without a urinary catheter is attempted.

### Psychometric evaluation of the ObsQoR-11T

To measure the convergent validity, the correlation between ObsQoR-11T score and global health VAS score was evaluated. Discriminant validity was tested by comparing the ObsQoR-11T score in two groups divided by the VAS (≥ 70 mm [good] vs. < 70 mm [poor]). To measure the construct validity, the correlation of continuous parameters with the ObsQoR-11T was evaluated. The ObsQoR-11T scores were compared in terms of education level, presence of comorbidities, parity groups, history of cesarean section, need for elective or emergency cesarean section, emergency category for emergency cesarean section, and type of anesthesia.

Cronbach's alpha, split-half reliability, and test–retest reliability were used to measure reliability. The test–retest reliability was analyzed in a subgroup of women who were asked to complete the questionnaire 60 min later (at 25 h), which was correlated to the 24-h results. The intra-class correlation coefficient was used to assess test–retest reliability. The floor and ceiling effects were calculated by determining if 15% of respondents received the greatest or lowest possible score. The recruitment rate, completion rate, and time taken to complete the scale were used to assess acceptability and feasibility (the investigator measured).

### Statistical analysis

The normal distribution of the continuous variables was tested using the Kolmogorov–Smirnov test. Measurement data are presented as mean ± standard deviation (SD), median (min–max) and categorical data are presented as frequency and percentage number (%). Differences in distribution were analysed by the Kruskal– Wallis test and Mann–Whitney U-test. Difference in distribution of categorical data was analysed by Fisher’s exact test and Chi-square test.

To achieve structural validity, confirmatory factor analysis (CFA) was performed.

It is suggested that sample size should be at least 10–15 times the number of items [11, 12]. According to Lacobucci, 50 can be sufficient for minimum sample size and 100 can be sufficient for maximum sample size [13]. ObsQoR-11 scale consisted of 4 dimensions and 11 items. It can be said that sample size of this study (160) is sufficient for CFA. CFA was performed using the Mplus 7 program [14]. To estimate the CFA model parameters, the Robust Maximum Likelihood estimation (MLR) method was used.

Correlations between the ObsQoR-11T items and VAS scores were measured using the Spearman rank (ρ) correlation coefficient. Internal consistency was measured using Cronbach’s α and split-half reliability. Test–retest reliability was measured using the intraclass correlation coefficient. All statistical analyses were performed using IBM SPSS statistics for Windows, version 25.0.; IBM Corp, Armonk, NY, USA. Differences were considered statistically significant when the *P*-value was < 0.05.

## Results

A total of 203 patients were screened for eligibility. Of these, 12 did not meet the inclusion criteria. The final sample consisted of 191 patients and there were no refusals (recruitment rate: 94%). After recruitment, five patients were excluded before the postoperative follow-up. A total of 186 patients completed the ObsQoR-11T after CD (completion rate: 97.38%). The mean time taken to complete the postoperative ObsQoR-11T scale was 123 ± 45 s for all patients, 121 ± 41 s for patients with elective CD, and 125 ± 43 s for patients with emergency CD (*P* = 0.173). Patient demographic characteristics are summarized in Table 1, medical characteristics and obstetric indications for CD are summarized in Table 2.

**Table 1:** Patient demographic characteristics

	All Cohort (n = 186)	Elective CD (n = 92)	Emergency CD (n = 94)	*P*-value
Maternal age (years), median (min–max)	28 (18–44)	28 (19–44)	28 (18–44)	0.735
*Parity, n (%)*				
0	28 (15%)	10 (10.9%)	18 (19.1%)	0.138
1	76 (40.8%)	36 (39.1%)	40 (42.6%)	
2	50 (26.8%)	32 (34.8%)	18 (19.1%)	
3	28 (15%)	12 (13%)	16 (17%)	
≥ 4	4 (2.1%)	2 (2.2%)	2 (2.1%)	
Weight (kg), median (min–max)	72 (54–127)	74 (58–127)	70 (54–105)	0.007*
Height (cm), median (min–max)	168 (150–176)	163.5 (150–176)	162 (155–170)	0.326
BMI (kg/m^2^), median (min–max)	26.97 (19.37–41.78)	27.85 (19.37–41.78)	26.31 (20.08–39.52)	0.006*
*Pre-existing medical conditions, n (%)*				
None	116 (62.4%)	68 (73.9%)	48 (51.1%)	0.001*
Yes	70 (37.6)	24 (26.1%)	46 (48.9%)	
*Gestation, n (%)*				
Single	182 (97.8%)	90 (97.8%)	92 (97.9%)	0.98
Multiple	4 (2.1%)	2 (2.2%)	2 (2.1%)	
*Education, n (%)*				
Elementary	52 (27.9%)	24 (26.1%)	28 (29.8%)	0.732
High school	78 (41.9%)	38 (41.3%)	40 (42.6%)	
University	56 (30.1%)	30 (32.6%)	26 (27.7%)	
Duration of surgery (min), median (min–max)	60 (30–100)	60 (30–100)	60 (30–70)	0.592
*Previous CD, n (%)*				
Yes	102 (54.8%)	58 (63%)	44 (46.8%)	0.019*
No	84 (45.1%)	34 (37%)	50 (53.2%)	
*Anaesthesia technique, n (%)*				
Single-shot spinal anesthesia	166 (89.2%)	82 (89.1%)	84 (89.4%)	0.573
General anesthesia	20 (10.7%)	10 (10.9%)	10 (10.6%)	
Length of hospital stay (day), median (min–max)	2 (1–5)	2 (1–3)	2.11 (2–5)	0.905

Data are presented with median (min–max), or numbers (percentages)

*CD* Cesarean delivery, *BMI* Body mass index

*Statistically significant at level of 0.05

**Table 2:** Patient medical characteristics and obstetric indications for CD (n = 186)

*Pre-existing medical conditions, n (%)*	
None	116 (62.4%)
Respiratory	6 (3.2%)
Cardiovascular	9 (4.8%)
Neurological	5 (2.6%)
Endocrine	20 (10.7%)
Haematological	11 (5.9%)
Musculoskeletal	6 (3.2%)
Psychiatric	3 (1.6%)
Others	10 (5.3%)
*Obstetric indication for CD, n (%)*	
Covid-19 Disease	4 (2.1%)
Pathological CTG	16 (8.6%)
Failure to progress	20 (10.7%)
Previous CD	102 (54.8%)
Breech	2 (1%)
Pre-eclampsia	6 (3.2%)
Uncontrolled DM	6 (3.2%)
Other maternal reasons	16 (8.6%)
Cephalopelvic disproportion	10 (5.3%)
Twins	4 (2.1%)

Data are presented with numbers (percentages)

*CD* Cesarean delivery, *CTG* Cardiotocography, *DM* Diabetes mellitus

**Fig. 2:**
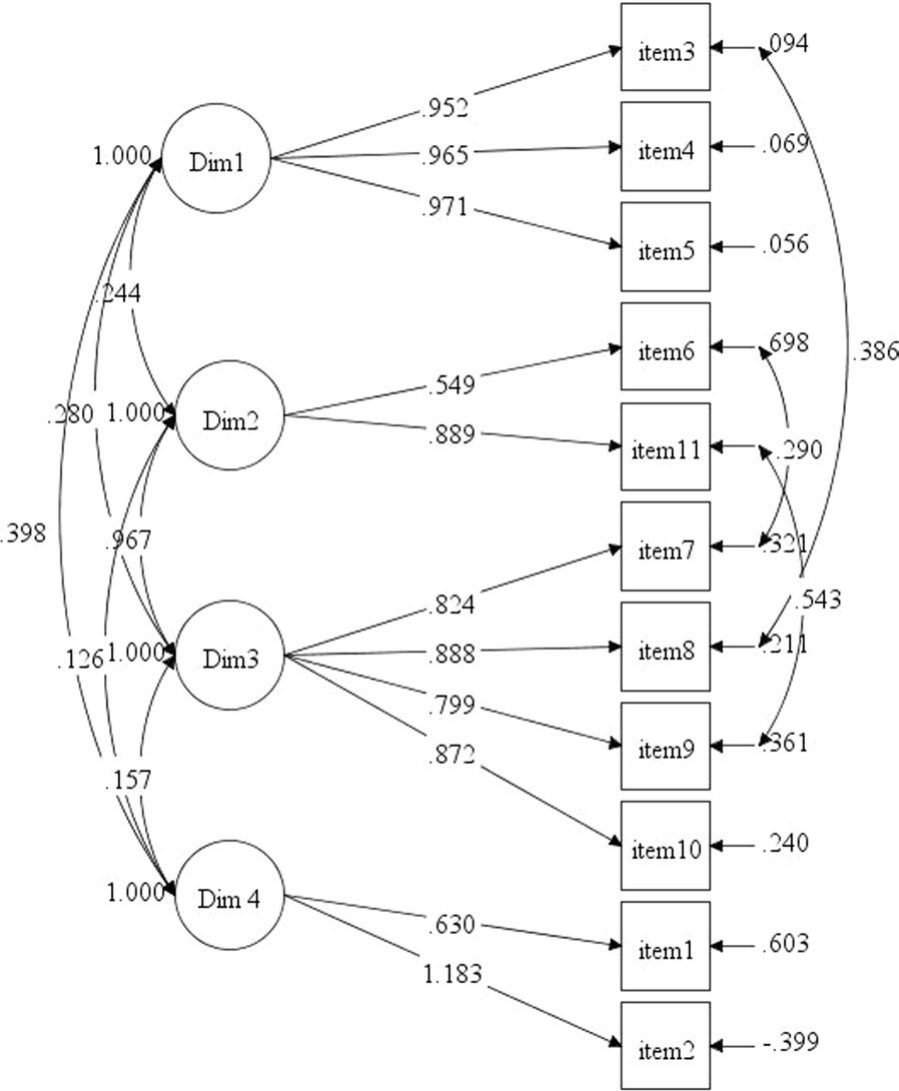
CFA model for ObsQoR-11T scale

The construct validity of ObsQoR-11T scale was determined via CFA analysis. After CFA analysis, the factor loadings and t values were examined and it was seen that all factor loadings were significant at the 0.05 level. The CFA model was given in Fig. 2. As seen in Fig. 2, all factor loadings were positive. The model-fit indexes were given in Table 3.

**Table 3:** Model-fit indexes for ObsQoR-11T scale

${\upchi }^{2}$	CFI	TLI	SRMR	RMSEA	90% CI RMSEA
69.439*	0.975	0.961	0.041	0.078	0.051–0.105

*ObsQoR-11T* Turkish version of Obstetric Quality of Recovery-11, *CFI* The comparative fit index, *TLI* Tucker-Lewis index, *SRMR* Standardized Root mean square residual, CI confidence interval, *RMSEA* Root mean square error of approximation

*P < 0.05

To determine model data fit, firstly the chi-square (χ^2^) test should be examined. The significance level of χ^2^ values greater than 0.05 indicate that model data fit provided. As seen in Table 3, significance of χ^2^ value was lower than 0.05. It can be said that the model data fit not achieved. However, χ^2^ test sensitive to sample size [15]. Therefore, beside χ^2^ test, other general goodness-of-fit indices (for e.g.CFI, TLI etc.) should be examined. In this study, in addition to χ^2^ statistic, CFI (the comparative fit index), TLI (Tucker-Lewis Index), SRMR (Standardized Root Mean Square Residual) and RMSEA (Root Mean Square Error of Approximation) values were examined. In order to obtain model data fit, SRMR index should be less than 0.08 [16] and RMSEA should be between 0.05 and 0.08 show an acceptable fit [17]. Moreover, CFI and TLI indices between 0.90 and 0.95 indicate acceptable fit [18]. As seen in Table 3, CFI is 0.975; TLI is 0.961; SRMR is 0.041 and RMSEA is 0.078. Al model fit indices proved that the model has an acceptable fit. As a result, it can be said that the construct validity of the ObsQoR-11T scale is provided.

To assess for convergent validity, we evaluated the correlation between the ObsQoR-11T and the VAS for recovery. The Spearman rho (ρ) correlation coefficient was 0.850 (95% CI 0.805 to 0.885) for all patients, 0.728 (95% CI 0.615–0.811) for patients with elective CD, and 0.868 (95% CI 0.808–0.910) for patients with emergency CD at the postoperative 24th hour following cesarean delivery (*P* < 0.0001). There was a strong correlation between the ObsQoR-11T and the VAS (correlation > 0.70). The individual item correlation to the VAS score is demonstrated in Table 4. Patients with a good or poor postoperative recovery, as indicated by a global health VAS ≥ 70 or < 70 mm, respectively, were compared to establish discriminant validity. The median [IQR] ObsQoR-11T score was significantly different between these groups (90 [83.1–94] vs. 71 [66–78.5]) (*P* < 0.0001). There were no statistically significant results in the correlation between the ObsQoR-11T score and the continuous variables (Table 5). In the comparison of the ObsQoR-11T score over categorical variables, only the difference between the emergency and elective CD groups (80 (41–104) vs. 83.3 (51–102)) was found to be significant (*P* < 0.05) (Table 6).

**Table 4:** Summary of correlations of ObsQoR-11T items to global health VAS (Spearmann’s *ρ*)

ObsQoR-11T Item	All Cohort (n = 186)		Elective CD (n = 92)		Emergency CD (n = 94)	
	Correlation to global health VAS score Spearman r (95% CI)	*P*-value	Correlation to global health VAS score Spearman r (95% CI)	*P*-value	Correlation to global health VAS score Spearman r (95% CI)	*P*-value
Moderate pain	0.50 (0.30–0.60)	< 0.0001	0.66 (0.53–0.76)	< 0.0001	0.32 (0.13–0.49)	< 0.0001
Severe pain	0.45 (0.33–0.56)	< 0.0001	0.57 (0.41–0.69)	< 0.0001	0.29 (0.09–0.47)	< 0.0001
Nausea or vomiting	0.40 (0.28–0.52)	< 0.0001	0.28 (0.08–0.46)	< 0.0001	0.49 (0.32–0.63)	< 0.0001
Dizzy	0.37 (0.24–0.49)	< 0.0001	0.28 (0.08–0.46)	< 0.0001	0.42 (0.24–0.57)	< 0.0001
Shivering	0.55 (0.44–0.64)	< 0.0001	0.48 (0.30–0.62)	< 0.0001	0.61 (0.47–0.72)	< 0.0001
Comfortable	0.30 (0.16–0.43)	< 0.0001	0.32 (0.12–0.49)	< 0.0001	0.28 (0.08–0.46)	< 0.0001
Mobilise independently	0.69 (0.61–0.76)	< 0.0001	0.68 (0.55–0.77)	< 0.0001	0.68 (0.55–0.78)	< 0.0001
Able–hold baby	0.69 (0.61–0.76)	< 0.0001	0.65 (0.51–0.75)	< 0.0001	0.71 (0.59–0.80)	< 0.0001
Able–nurse/feed baby	0.60 (0.50–0.68)	< 0.0001	0.64 (0.50–0.75)	< 0.0001	0.55 (0.39–0.68)	< 0.0001
Able to take care of personal hygiene	0.65 (0.57–0.73)	< 0.0001	0.56 (0.40–0.69)	< 0.0001	0.73 (0.62–0.81)	< 0.0001
Feeling in control	0.56 (0.45–0.65)	< 0.0001	0.51 (0.34–0.65)	< 0.0001	0.62 (0.48–0.73)	< 0.0001

*ObsQoR-11T* Turkish version of Obstetric Quality of Recovery -11, *CD* Cesarean delivery, *VAS* Visual analogue scale, *CI* Confidence interval

**Table 5:** Summary of correlations of clinical characteristics to ObsQoR-11T score for continuous variables

Clinical characteristic	All Cohort (n = 186)		Elective CD (n = 92)		Emergency CD (n = 94)	
	Correlation to ObsQoR-11T Spearman r (95% CI)	*P*-value	Correlation to ObsQoR-11T Spearman r (95% CI)	*P*-value	Correlation to ObsQoR-11T Spearman r (95% CI)	*P*-value
Weight	0.18 (0.03–0.31)	0.11	0.09 (− 0.13 to 0.28)	0.46	0.19 (− 0.02 to 0.38)	0.10
Height	0.19 (0.05–0.33)	0.08	0.19 (− 0.02 to 0.38)	0.08	0.12 (− 0.09 to 0.32)	0.27
Maternal age	− 0.03 (− 0.18 to 0.11)	0.75	− 0.00 (− 0.21 to 0.20)	0.98	− 0.02 (− 0.22 to 0.18)	0.86
Duration of surgery	0.12 (− 0.03 to 0.26)	0.29	0.10 (− 0.11 to 0.30)	0.39	0.12 (− 0.08 to 0.31)	0.26
Pre Hbg	− 0.01 (− 0.15 to 0.14)	0.95	− 0.11 (− 0.31 to 0.10)	0.31	0.06 (− 0.15 to 0.26)	0.59
Post Hbg	− 0.03 (− 0.17 to 0.12)	0.80	− 0.06 (− 0.26 to 0.15)	0.57	− 0.07 (− 0.30 to 0.13)	0.49
Change in Hbg	0.00 (− 0.14 to 0.14)	0.99	− 0.03 (− 0.24 to 0.17)	0.76	− 0.01 (− 0.21 to 0.20)	0.96
LOS (day)	− 0.12 (− 0.26 to 0.03)	0.27	− 0.23 (− 0.42 to − 0.030)	0.03	0.05 (− 0.15 to 0.25)	0.62
BMI	0.13 (− 0.02 to 0.27)	0.26	0.000 (− 0.20 to 0.20)	0.99	0.20 (0–0.39)	0.07

*ObsQoR-11T* Turkish version of obstetric quality of recovery -11 *CD* Cesarean delivery, *CI* Confidence interval, *Hbg* Hemoglobin, *LOS* Length of hospital stay, *BMI* Body mass index

**Table 6:** Comparison of the ObsQoR-11T score over categorical variables (n = 186)

	Total ObsQoR-11T Score median (min–max)	*P*-value
*Parity*		
0	80 (41–100)	0.224
1	80 (51–97)	
2	83 (51–101)	
3	78.5 (59–104)	
≥ 4	86 (84–88)	
*Pre-existing medical conditions,*		
None	81 (41–104)	0.102
Yes	80 (51.6–96)	
Gestation		
Single	80 (41–104))	0.640
Multiple	77.9 (75–83)	
*Education*		
Elementary	78.5 (51.6–104)	0.620
High school	83 (41–101)	
University	80 (65–100)	
*Previous CD*		
Yes	80 (51–104)	0.693
No	80 (41–100)	
*Cesarean delivery type*		
Elective CD	83.3 (51–102)	0.010
Emergency CD	80 (41–104)	
*Anaesthesia technique*		
Single-shot spinal anesthesia	80 (41–102)	0.152
General anesthesia	86.5 (67–104)	

*ObsQoR-11T* Turkish version of Obstetric Quality of Recovery -11, *CD* Cesarean delivery

Internal consistency measured using Cronbach’s alpha was 0.822 for all patients; 0.821 in patients delivering by elective CD, and 0.814 in those delivering by emergency CD. The inter-item correlation matrix for the ObsQoR-11T is outlined in Table 7. Inter-item correlations were mostly at r > 0.15 (82%) for all patients, r > 0.15 (85%) for patients with elective CD, r > 0.15 (75%) for patients with emergency CD, a good indicator of consistency. Split-half reliability with the Spearman-Brown adjustment (which measures the extent to which all parts of the test contribute equally to the desired measurement) was 0.708 for all patients, 0.697 for patients with elective CD, 0.703 for patients with emergency CD, implying an equal contribution from all items. The test–retest reliability of the ObsQoR-11T items was r > 0.6 in 82% of items and ≥ 0.45 in the remaining items (no. 4 and 5) for all patients, r > 0.6 in 64% of items for patients with elective CD, r > 0.6 in 82% of items for patients with emergency CD, suggesting adequate repeatability and reliability (Table 8). The percentage of women who achieved the highest and lowest possible ObsQoR-11 scores at 24 h was 0% (n = 0/186). Therefore, no floor or ceiling effects of the scoring tool were demonstrated. The ObsQoR-11 T scores were negatively skewed. The level of skewness was − 0.515 for all patients, − 0.610 for patients with elective CD, − 0.458 for patients with emergency CD at 24 h postoperatively, indicating that the majority of the ObsQoR-11T scores were greater than 55 points.

**Table 7:** Inter-item correlation matrix for ObsQoR-11T following caesarean delivery

	ObsQoR-11T item number	Total ObsQoR-11T score	1	2	3	4	5	6	7	8	9	10	11
All Cohort (n = 186)	1	0.51**	–										
	2	0.60**	0.59**	–									
	3	0.52**	0.02	0.04	–								
	4	0.49**	0.02	0.03	0.78**	–							
	5	0.65**	0.17	0,16	0.72**	0.63**	–						
	6	0.41**	0.14	0.09	0.13	0.23*	0.22*	–					
	7	0.71**	0.38**	0.42**	0.12	0.17	0.34**	0.37**	–				
	8	0.74**	0.24*	0.28**	0.43**	0.36**	0.41**	0.26*	0.54**	–			
	9	0.64**	0.12	0.31**	0.29**	0.25*	0.37**	0.30**	0.39**	0.64**	–		
	10	0.75**	0.23*	0.43**	0.32**	0.26*	0.39**	0.22*	0.58**	0.59**	0.43**	–	
	11	0.61**	0.07	0.18	0.25*	0.22*	0.32**	0.30**	0.49**	0.46**	0.45**	0.64**	–
Elective CD (n = 92)	1	0.58**	–	–	–	–	–	–	–	–	–	–	–
	2	0.56**	0.52**	–	–	–	–	–	–	–	–	–	–
	3	0.50**	0.25*	0.22*	–	–	–	–	–	–	–	–	–
	4	0.46**	0.07	− 0.01	0.47**	–	–	–	–	–	–	–	–
	5	0.61**	0.36**	0.23*	0.27**	0.34**	–	–	–	–	–	–	–
	6	0.31**	0.08	− 0.08	0.07	0.33**	0.01	–	–	–	–	–	–
	7	0.69**	0.28**	0.33**	0.01	0.13	0.29**	0.23*	–	–	–	–	–
	8	0.76**	0.30**	0.19	0.32**	0.34**	0.41**	0.15	0.643**	–	–	–	–
	9	0.70**	0.22*	0.21*	0.20	0.33**	0.43**	0.16	0.457**	0.769**	–	–	–
	10	0.79**	0.31**	0.41**	0.33**	0.20	0.32**	0.18	0.627**	0.560**	0.469**	–	–
	11	0.71**	0.12	0.24*	0.18	0.26*	0.27*	0.33**	0.626**	0.500**	0.524**	0.815**	–
Emergency CD (n = 94)	1	0.39**	–	–	–	–	–	–	–	–	–	–	–
	2	0.39**	0.61**	–	–	–	–	–	–	–	–	–	–
	3	0.50**	0.04	− 0.17	–	–	–	–	–	–	–	–	–
	4	0.52**	− 0.01	− 0.07	0.59**	–	–	–	–	–	–	–	–
	5	0.70**	0.05	− 0.03	0.69**	0.63**	–	–	–	–	–	–	–
	6	0.58**	0.15	0.14	0.17	0.39**	0.37**	–	–	–	–	–	–
	7	0.68**	0.38**	0.37**	0.11	0.12	0.37**	0.32**	–	–	–	–	–
	8	0.74**	0.13	0.17	0.32**	0.28**	0.40**	0.36**	0.47**	–	–	–	–
	9	0.56**	− 0.09	0.13	0.20	0.10	0.37**	0.26*	0.27**	0.59**	–	–	–
	10	0.78**	0.18	0.28**	0.24*	0.37**	0.46**	0.39**	0.60**	0.63**	0.30**	–	–
	11	0.67**	0.09	0.03	0.30**	0.20	0.44**	0.32**	0.44**	0.49**	0.46**	0.65**	–

*ObsQoR-11T* Turkish version of Obstetric Quality of Recovery-11, *CD* Cesarean delivery

*Correlation is significant at the level 0.05 level (2-tailed)

** Correlation is significant at the level 0.01 level (2-tailed)

**Table 8:** Intra-class correlations for ObsQoR-11T items

ObsQoR-11T item	All Cohort (n = 186)	Elective CD (n = 92)	Emergency CD (n = 94)
Item no. 1: Moderate pain	0.69	0.62	0.78
Item no. 2: Severe pain	0.64	0.61	0.70
Item no. 3: Nausea or vomiting	0.61	0.58	0.66
Item no. 4: Dizzy	0.45	0.32	0.55
Item no. 5: Shivering	0.48	0.31	0.50
Item no. 6: Comfortable	0.67	0.47	0.87
Item no. 7: Mobilise independently	0.72	0.75	0.69
Item no. 8: Able to hold baby	0.74	0.78	0.70
Item no. 9: Able to nurse/feed baby	0.76	0.72	0.80
Item no. 10: Able to take care of personal hygiene	0.84	0.85	0.84
Item no. 11: Feeling in control	0.78	0.77	0.80
ObsQoR-11T score	0.79	0.76	0.84

ObsQoR-11T 24 versus 25 h scores. Spearman correlation > 0.6 in 82% of items all items indicates good reliability of the instrument in all cohort

## Discussion

The results of our study showed that the ObsQoR-11T was a valid, reliable, clinically convenient, and suitable scale for measuring the quality of postoperative recovery after both elective and non-elective CD in the Turkish-speaking population. In addition to being an ideal scale for evaluating convergent validity with the ObsQoR-11T, the global health VAS is the most frequently used scale and the gold standard. The QoR-40 scale, which is the source of the ObsQoR-11 scale, lacks content validity for obstetric recovery because it does not include items pertaining to care of a baby. Especially at the postoperative 24th hour, a strong correlation was found between the ObsQoR-11T scores and VAS scores (r = 0.850). This achieved the > 0.6 criterion for health rating scales, demonstrating that the ObsQoR-11T has excellent convergent validity, and more strongly than the original ObsQoR-11 (r = 0.53) [5]. Regarding the surgical types including general surgery, orthopedics, and otolaryngological surgeries, the QoR-40T scale was evaluated on the 3rd postoperative day in terms of convergent validity and the result was r = 0.468 [4]. As such, the ObsQoR-11T is better than the QoR-40T in terms of convergent validity.

The discriminant validity of the study was confirmed by comparing women with good or poor postoperative recovery, as indicated by the global health VAS score [19]. In the original study [5], the good versus poor recovery median values (100 vs. 87) according to the VAS score after the elective CD were found to be 97 versus 64. In the validation study after non-selective CD, it was found to be 90 versus 71 in the ObsQoR-11T [20]. While discriminatory validity was achieved in all three studies, the difference between the scores is due to the difference between the postoperative recovery procedures of the centers. Moreover, overall floor and ceiling effects were absent. Hence, it is feasible to use ObsQoR-11T after CD.

The construct validity was determined by conducting CFA, considering the data collected from 186 participants and the original dimensions and items. Turkish adaptation of the ObsQoR-11 scale’s original structure was confirmed and structural validity was ensured.

In our study, a statistical difference was observed between the global ObsQoR-11T scores (74 vs. 82) after emergency and elective CD. While the pregnant women for elective CDs were able to psychologically prepare themselves and their expectations, women who underwent emergency CDs did not have any time for preparation. This may explain the difference in the ObsQoR-11T scores during the recovery period. Moreover, complications are naturally more likely to occur in emergency CDs and this may have decreased the quality of the postoperative recovery. In our study, no correlations were found between other recorded demographic and surgical data, and the ObsQoR-11T. In studies based on the specific evaluation of these variables in the future, the ObsQoR-11T score should be evaluated. Unlike the QoR-40 T study, correlations of the ObsQoR-11T with these variables could not be shown. This demonstrates that the postoperative recovery period after CD is a more unique and complex process than other surgeries.

Cronbach's alpha and split-half reliability were 0.82 and 0.70, respectively, and comparable with those reported for the original ObsQoR-11 and QoR-40 T [4, 5]. Cronbach's alpha was more than 0.7 which is above the recommended criterion [21]. Internal consistency was also tested by inter-item correlation, with high values indicating strong item correlation within the instrument. The correlation coefficients between the items and the global ObsQoR-11T scores were between 0.41 and 0.75, and the lowest coefficient value was related to the 6^th^ item, while the highest coefficient value was related to the 10^th^ item. In the original study, the lowest values were found in items 8 and 9, while the highest coefficient value was found in item 1 [5]. In both the original study and our study, negative correlation values were not obtained in the inter-item correlation matrix. These results were enough to confirm that the ObsQoR-11T possesses adequate reliability.

There is no consensus on the timing of test repetition in the QoR studies [21, 22]. To set a period long enough not to remember the answers given after 24 h, but short enough not to deviate significantly from the health status at 24 h, we also performed a retest at the 25th hour, similar to previous studies. The test–retest reliability was excellent.

The presence of questions containing both negative and positive expressions in the same questionnaire causes difficulties in the psychometric evaluation process. While in the first five questions of the questionnaire, the 11-point Likert scale starts from 10, it starts from 0 in the second section consisting of six questions. This sudden change resulted in confusion. The person administering the questionnaire may need to provide guidance with a proactive attitude to prevent confusion.

While we typically determine 0 as no pain and 10 as the most severe pain in the postoperative pain assessment using the VAS or NRS, 10 expresses the most pain-free situation in the pain questions, which are the first two questions of the ObsQoR-11 scale. Having two separate questions for pain assessment also caused confusion. Our patients described the pain as ‘‘tolerable pain’’ for moderate pain and ‘‘terrible pain’’ for severe agonizing pain.

There are some limitations to our study. Patients with the most severe illnesses, who would receive the worst scores if they were unable to consent or complete the survey within 24 h, as well as those from other cultures who needed assistance understanding written Turkish or those with less education, could have been exclude of the study. To measure responsiveness in the validation studies of the quality of recovery scales, the same questionnaire was applied both preoperatively and postoperatively, and Cohen effect size and standardized response mean measurements were performed [23, 24]. As there were questions such as “I can hold my baby without help” and “I can breastfeed my baby without help” in the questionnaire, the preoperative ObsQoR-11 evaluation was not completely objective. Moreover, unlike the original study, the preoperative questionnaire was not applied since non-elective cases were also included in our study. The other limitation of our study was that it was single-centered. In addition, if the baby is taken to the neonatal ICU (NICU) after birth, questions 8 and 9 about the baby may not be possible to answer, therefore ObsQoR-11T measurement will result in a different score if the baby is admitted to the NICU.

## Conclusions

In conclusion, current study evaluated the Turkish version of the ObsQoR‐11 scoring tool to measure QoR on the first postoperative day after CD in a single centre. In terms of validity, reliability, clinical acceptability, and feasibility, the ObsQoR-11T performed well. The questionnaire may be used to assess postoperative recovery after CD as a standardized patient-reported outcome measure. More research is needed to validate this tool in spontaneous or assisted vaginal deliveries, as well as in patients with babies admitted to the NICU, taking their ability to nurse/feed/hold the baby into account.

## Data Availability

Data are presented in the manuscript.
